# Anterior Disc Displacement in a Young Patient With Stage II Osteoarthritis: Diagnostic and Therapeutic Approach. A Case Report

**DOI:** 10.1155/crid/2040786

**Published:** 2026-09-30

**Authors:** Daniela Chamba-León, Madeleyne Priscila Serrano Toledo, Juan Diego Sabay-Aldas, Kevin Ochoa Suarez, Wilson Daniel Bravo Torres

**Affiliations:** ^1^ Department of Oral Rehabilitation Faculty of Dentistry University of Cuenca Cuenca Ecuador ucuenca.edu.ec; ^2^ Faculty of Dentistry-GIRO Group University of Cuenca Cuenca Ecuador ucuenca.edu.ec

**Keywords:** anterior disc displacement, arthrocentesis, case report, temporomandibular joint, temporomandibular joint osteoarthritis

## Abstract

Anterior disc displacement without reduction can cause acute functional limitations and may coexist with inflammatory and early degenerative changes in young patients. We report a 22‐year‐old woman with right preauricular pain, a closed lock, and a maximum unassisted interincisal opening of 19 mm. Cone‐beam computed tomography and magnetic resonance imaging confirmed Stage II osteoarthritis of the right temporomandibular joint, right anterior disc displacement without reduction with limited opening, left anterior disc displacement with reduction, and bilateral joint effusion consistent with synovitis. Hormonal testing showed values discordant with the expected follicular‐phase ranges after recent discontinuation of injectable contraception; this finding was interpreted as a potential modulatory factor rather than evidence of causation. Management included bilateral temporomandibular joint arthrocentesis, intra‐articular administration of betamethasone and sodium hyaluronate, a stabilization splint, pharmacological therapy, physical therapy, and behavioral modification. Pain decreased markedly within the first week. At the 3‐month follow‐up, maximum unassisted mouth opening was 42 mm, with a straight opening path, pain‐free function, and resolution of the right masseter spasm. This case highlights the value of early imaging‐based diagnosis and individualized multimodal management in young patients with concomitant mechanical, inflammatory, and early degenerative temporomandibular joint changes.

## 1. Introduction

Pain involving the temporomandibular joint (TMJ) or the masticatory muscles, impaired mandibular movement, and joint sounds are common manifestations of temporomandibular disorders (TMDs), which may interfere with oral function and quality of life [1–5]. The Diagnostic Criteria for Temporomandibular Disorders (DC/TMD) provide a standardized two‐axis framework that combines validated clinical criteria for common pain‐related and intra‐articular disorders with an assessment of psychosocial and behavioral factors relevant to pain‐related disability and clinical management [1]. Prevalence estimates vary according to the population and diagnostic methodology. A 2024 meta‐analysis estimated a global prevalence of approximately 34%, with higher rates among women and adults aged 18–60 years and a regional estimate of 47% in South America [6, 7]. In Ecuador, TMDs were identified in 65.8% of adults evaluated at the clinics of the University of Cuenca, highlighting their relevance within the local clinical setting [8].

Among intra‐articular TMDs, anterior disc displacement without reduction disrupts normal condyle–disc translation and may result in painful restriction of mouth opening or an acute closed lock [1, 4]. In young patients, this mechanical alteration may coexist with joint effusion and early osseous changes, requiring complementary imaging to characterize both soft‐ and hard‐tissue involvement [2]. Although hormonal influences on TMJ tissues have been investigated in relation to the greater prevalence of TMDs among women, current evidence supports a potential modulatory role rather than a direct causal relationship [1, 3]. The clinical relevance of this case lies in the simultaneous identification, in a 22‐year‐old woman, of right anterior disc displacement without reduction with limited opening, Stage II osteoarthritis, bilateral joint effusion consistent with synovitis, and contralateral disc displacement with reduction. This report describes the integration of clinical, cone‐beam computed tomography (CBCT), and magnetic resonance imaging (MRI) findings, together with the patient′s 3‐month clinical course following individualized multimodal management.

## 2. Case Presentation

A 22‐year‐old female patient presented with pain on the right side of her face that had persisted for 1 month and had progressively worsened over the preceding weeks. The patient reported continuous pain in the right preauricular region with an intensity of 6/10 on a visual analog scale (VAS) at rest and 8/10 during mastication. The pain radiated to the ipsilateral masseter muscle and was exacerbated by eating. The patient reported occasional use of paracetamol, which provided partial and temporary symptom relief.

Regarding her relevant history, the patient reported no significant medical, dental, or family history. She denied previous facial or mandibular trauma, rheumatic or joint diseases, previous treatment for TMDs, or use of an occlusal splint. She also reported excessive gum chewing during classes, approximately 60 pieces per week. She also reported the monthly use of injectable hormonal contraception (Femgyl, consisting of 50 mg of norethisterone enanthate and 5 mg of estradiol valerate) for 1 year, with the last injection administered approximately 2 months before the onset of symptoms.

### 2.1. Clinical Examination

The maximum interincisal opening without assistance was 19 mm and was accompanied by pain. Assisted opening did not result in a significant increase, suggesting a mechanical restriction.

During opening, mandibular deflection to the right was evident, and no joint sounds were detected on auscultation.

Lateral palpation of the right TMJ was painful. Pain was also elicited on palpation of the right masseter muscle, with associated muscle spasm. Forced protrusion tests were not performed because pain worsened during the evaluation.

The patient exhibited bilateral Class I occlusion, with no evidence of tooth wear or clinically apparent occlusal interference.

### 2.2. Imaging Studies

#### 2.2.1. CBCT

Sagittal and coronal views of the right TMJ revealed condylar cortical erosion of approximately 2.5 mm, which was consistent with initial degenerative changes. Upon mouth opening, the right condyle exhibited hypomobility while remaining essentially within the glenoid fossa (Figure 1).

**Figure 1:**
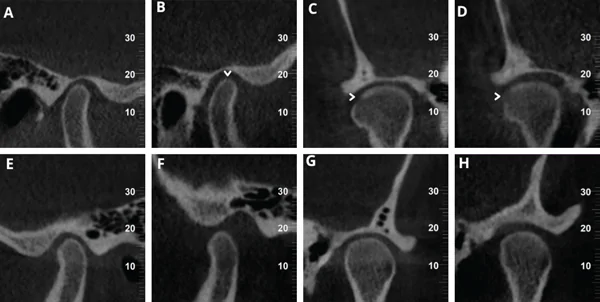
Cone‐beam computed tomography (CBCT) images of the temporomandibular joints. (A–D) Right TMJ (sagittal and coronal images in the closed and open positions of the mouth). Focal cortical erosion is evident on the anterosuperior surface of the right mandibular condyle, visible in the sagittal and coronal planes in both the closed and open positions of the mouth (arrowheads). On the coronal images, the cortical defect measures approximately 2.5 mm at its greatest dimension (arrowheads). In the open position of the mouth, condylar hypomobility is evident, with the condyle remaining within the mandibular fossa without reaching the articular eminence. No defined osteophytes or advanced condylar deformity are observed. The findings are consistent with early degenerative changes, corresponding to Stage II osteoarthritis. (E–H) Left TMJ (sagittal and coronal images in the closed and open positions of the mouth). The mandibular condyle shows preserved morphology and continuous cortical bone, without erosions or evident degenerative changes. In the closed position of the mouth, slight condylar retroversion is observed within the mandibular fossa. With the mouth opened maximally, the condyle clearly translates towards the articular eminence, without completely reaching its most superior point.

#### 2.2.2. MRI

Bilateral T1‐ and T2‐weighted MR imaging, performed in both the closed and open positions of the mouth, revealed anterior disc displacement without reduction in the right TMJ and no morphological alterations of the articular disc. In the left TMJ, the disc was anteriorly displaced in the closed mouth position but was recaptured during opening, restoring the physiological disc–condyle relationship and confirming anterior disc displacement with reduction (Figure 2).

**Figure 2:**
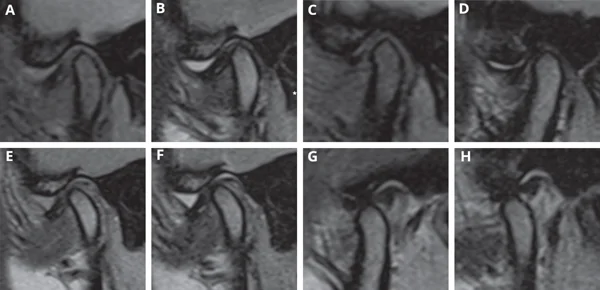
Magnetic resonance images of the temporomandibular joints (sagittal T1‐ and T2‐weighted images in the closed and open positions of the mouth). (A–B) Right TMJ in the closed position of the mouth (T1–T2): Anterior displacement of the articular disc relative to the condylar head is observed, with the disc positioned anterior to the 12 o′clock position. On T2‐weighted imaging, a hyperintense signal in the superior articular compartment is evident, consistent with synovial effusion. (C–D) Left TMJ in the closed position of the mouth (T1–T2): The articular disc is located anterior to the mandibular condyle, which is positioned posteriorly within the glenoid fossa. A hyperintense signal is identified in the superior articular compartment on the T2‐weighted image, consistent with mild synovial effusion. (E–F) Right TMJ in the open position of the mouth (T1–T2): Anterior displacement of the disc persists without recapture during mandibular translation, consistent with anterior disc displacement without reduction. Limited condylar translation is observed. Synovial effusion persists on T2‐weighted imaging. (G–H) Left TMJ in the open position of the mouth (T1‐ and T2‐weighted images): The articular disc has been recaptured and is correctly interposed between the condyle and the eminence, restoring the disc–condyle relationship. No significant structural changes are observed.

On the T2‐weighted images, bilateral synovial effusion was identified and was more pronounced in the right joint (Figure 2).

### 2.3. Complementary Studies

Serum hormonal tests and measurement of salivary cortisol concentration were performed as part of the comprehensive assessment. Salivary cortisol concentration was within the normal range, whereas the serum hormonal profile revealed a prolactin concentration of 24.26 ng/mL, an estradiol (E2) concentration of 252.5 pg/mL, a follicle‐stimulating hormone (FSH) concentration of 1.82 IU/L, a luteinizing hormone (LH) concentration of 7.12 IU/L, and a testosterone concentration of 0.07 ng/mL.

According to the date of the last menstruation, the patient was in the follicular phase at the time of sampling; however, the values of estradiol and FSH were not within the expected ranges for this phase, indicating a hormonal profile discordant with the physiological follicular pattern.

Given the marked limitation in mouth opening, the differential diagnosis included disc displacement with reduction with intermittent locking and capsular fibrosis. Intermittent locking was considered less likely because the patient presented with persistent restriction, and MRI demonstrated anterior disc displacement without recapture during opening. Capsular fibrosis was also considered less likely because the symptoms had developed over a relatively short period and there was no history of chronic mandibular restriction.

The definitive diagnosis was Stage II osteoarthritis of the right TMJ, right anterior disc displacement without reduction with limited opening, left anterior disc displacement with reduction, and bilateral synovitis.

At the time of diagnosis, the short‐term prognosis for pain control and functional recovery was considered favorable because the degenerative changes were at an early stage, and no advanced condylar deformity was observed.

The chronological sequence of the clinical presentation, diagnostic assessment, treatment, and follow‐up is summarized in Table 1.

**Table 1:** Timeline of the clinical presentation, diagnostic assessment, treatment, and follow‐up.

Time point	Clinical event
Approximately 1 month before the initial consultation	Onset of continuous right preauricular pain, followed by progressive restriction of mandibular opening and the development of a closed‐lock episode.
Day 0	Initial consultation. The patient reported right preauricular pain rated 6/10 at rest and 8/10 during mastication. Maximum unassisted interincisal opening was 19 mm, with right‐sided mandibular deflection and no significant increase during assisted opening.
Day 1	Cone‐beam computed tomography was performed. The examination revealed focal cortical erosion of the anterosuperior surface of the right mandibular condyle, measuring approximately 2.5 mm, together with restricted condylar translation. These findings were consistent with early degenerative changes corresponding to Stage II osteoarthritis.
Day 3	Magnetic resonance imaging of both temporomandibular joints was performed in the closed and open mouth positions. The examination confirmed right anterior disc displacement without reduction with limited opening, left anterior disc displacement with reduction, and bilateral joint effusion, which was more pronounced on the right side.
Day 7	Bilateral temporomandibular joint arthrocentesis was performed using a two‐needle technique, followed by intra‐articular administration of betamethasone and sodium hyaluronate. Adjunctive pharmacological treatment, occlusal splint therapy, physical therapy, and behavioral interventions were initiated.
1 week after treatment	One‐week follow‐up. The patient reported a marked reduction in joint pain and early improvement in mandibular function. No adverse events were observed.
1 month after treatment	One‐month follow‐up. Maximum unassisted mouth opening increased to 42 mm, with a straight opening path, absence of pain during opening and mastication, and resolution of the right masseter muscle spasm.
3 months after treatment	Three‐month follow‐up. The functional improvement was maintained, with no recurrence of pain or closed lock and no adverse or unanticipated events.

### 2.4. Therapeutic Management

Based on the diagnoses of right anterior disc displacement without reduction, Stage II osteoarthritis of the right TMJ, and bilateral synovitis, an individualized multimodal treatment approach was implemented to control intra‐articular inflammation, restore mandibular mobility, and improve joint biomechanical stability [4, 9–12].

Bilateral TMJ arthrocentesis was performed with a two‐needle technique involving the superior joint compartment. In each joint, the two needles were used to establish a continuous irrigation system. Lavage was performed with sterile 0.9% saline until the effluent became clear, requiring approximately 70 mL for the right TMJ and 50 mL for the left TMJ. This procedure decreased intra‐articular pressure and facilitated the removal of inflammatory mediators [11, 13]. Following lavage, 0.5 mL of betamethasone solution (commercial formulation: 6 mg/2 mL betamethasone; administered dose: 1.5 mg) and 0.5 mL of sodium hyaluronate (commercial formulation: 25 mg/2.5 mL; administered dose: 5 mg) were sequentially injected into each TMJ, resulting in a total intra‐articular volume of 1 mL per joint [14–16]. This combined approach has been demonstrated to be effective in reducing pain and improving mandibular function in patients with TMDs [17, 18].

As an orthotic adjunct, a maxillary occlusal stabilization splint with an esthetic anterior extension was fabricated, increasing the occlusal vertical dimension (OVD) by 2 mm. The patient was instructed to wear the appliance during the daytime for 3 months.

Pharmacological management included the administration of a nonsteroidal anti‐inflammatory drug (NSAID) alongside a muscle relaxant, specifically a combination of 15 mg meloxicam and 4 mg pridinol mesylate (Bienexflex), every 12 h for 5 days. Additionally, Piascledine (avocado and soybean oil unsaponifiables) was administered at a dose of 300 mg (one capsule) once daily for 3 months as a chondroprotective adjuvant [19].

To complement pharmacological treatment, the patient was referred to physical therapy, which included therapeutic electrostimulation to reduce muscle hyperactivity and promote functional recovery [9, 12]. Physical therapy sessions were initiated 1 month after arthrocentesis. Weekly sessions were performed during the following month, followed by monthly sessions until the third month after arthrocentesis.

Behavioral changes were recommended, which included consumption of a soft diet, immediate suspension of the habit of chewing gum, and limiting wide mandibular opening [12].

### 2.5. Clinical Evaluation

Clinical evaluations were performed at 1 week, 1 month, and 3 months after the procedure. At the 1‐week evaluation, the patient reported a significant decrease in joint pain, and early functional improvement was evident.

After 1 month, the patient showed progressive improvement in the range of mandibular opening and a marked reduction in symptoms, with no recurrence of closed lock:

• Maximum unassisted mouth opening of 42 mm.
• A straight opening path.
• Absence of pain during mouth opening and mastication.
• Resolution of the masseter spasm.

The clinical course was favorable, and complete functional recovery was achieved during the follow‐up period (Figure 3).

**Figure 3:**
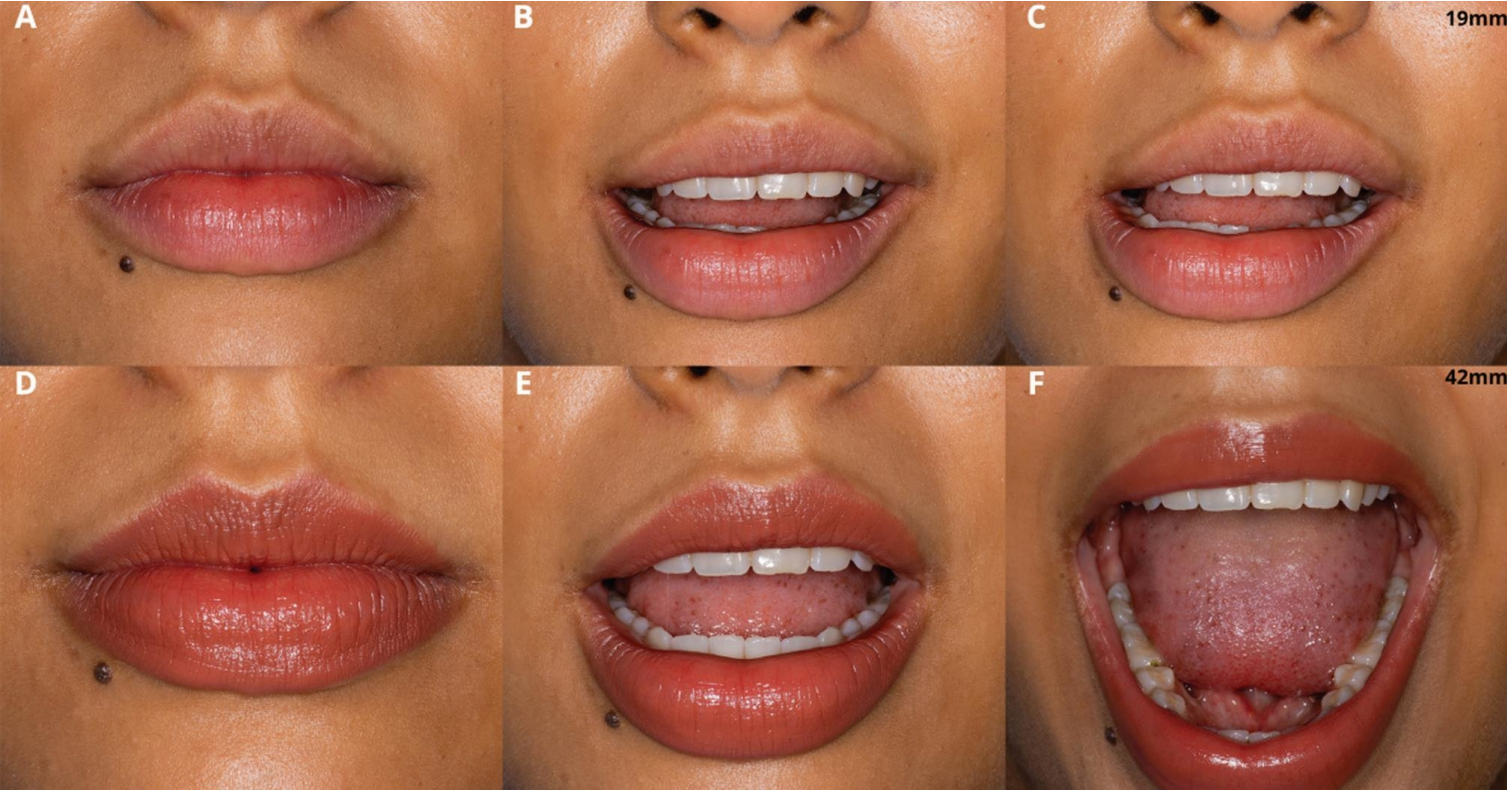
Clinical evaluation of mandibular opening before and after treatment. (A–C) Initial evaluation showing limited mandibular opening, with a maximum unassisted interincisal opening of 19 mm. (D–F) Clinical examination at the 3‐month follow‐up showing a straight opening trajectory and a maximum interincisal opening of 42 mm. The values displayed in Panels C and F correspond to the interincisal measurements recorded clinically during the respective examinations.

Adherence to splint use, pharmacological treatment, and behavioral recommendations was assessed through patient self‐report at each follow‐up visit. Physical therapy was provided at the head and neck physical therapy unit of the same medical center; therefore, attendance and completion of the prescribed sessions were corroborated through institutional records and communication with the physical therapy team. No adverse or unanticipated events were reported during the follow‐up period, and the patient tolerated the therapeutic approach well.

## 3. Discussion

The interplay among mechanical factors, intra‐articular inflammatory responses, and potential systemic modulators contributes to the complex clinical presentation of TMDs. In the present case, the coexistence of closed lock, Stage II osteoarthritis, and bilateral synovial effusion in a young patient illustrates that the structural and inflammatory changes may occur simultaneously during the early stages of the clinical presentation [2, 20].

Anterior disc displacement without reduction is clinically associated with substantial limitations in mandibular opening, deflection towards the affected side, and the absence of noises in the joint, characteristics that are used to describe the so‐called “closed lock” [10, 20]. Among young adults, degenerative condylar changes can be identified even in the early stages of symptomatic disc displacement [2, 21]. However, the presence of initial bone alterations does not necessarily imply irreversible structural progression but may reflect adaptive phenomena or remodeling within an altered biomechanical environment [21].

Articular effusion identified on MR images is a frequent finding in patients experiencing pain. Hyperintensity on T2‐weighted images is associated with arthralgia and functional pain, although the association between the presence of intra‐articular fluid and symptomatology is not absolute [22, 23]. In the present case, the more pronounced effusion in the symptomatic TMJ supported the clinical assessment without implying a direct causal relationship between disc displacement and synovitis [22].

Female sex is an important biological factor in TMD research. Estrogen receptors (ER*α* and ER*β*) have been identified in the condylar cartilage, articular disc, and synovial tissues of the TMJ, and estrogen signaling may influence cartilage metabolism and inflammatory responses [3]. However, these mechanisms are complex and do not demonstrate that hormonal variation causes a specific TMD [3]. The higher frequency of joint involvement reported in young women, therefore, supports the consideration of endocrine modulation as one of several potential susceptibility factors [24].

In the present case, the patient had discontinued injectable hormonal contraception 2 months prior to the onset of symptoms. It is possible that during the period when the hypothalamic–pituitary–ovarian axis was restored, transient estrogen fluctuations occurred, which could have influenced nociceptive sensitivity or the local inflammatory response of the condyle–disc complex. However, the hormonal profile was based on a single measurement with no baseline or serial measurements available. Therefore, although biological evidence supports a possible relationship between estrogen and TMJ inflammatory and degenerative processes, contraceptive withdrawal cannot be established as a direct causal factor [3]. In this case, it should be interpreted only as a possible modulator within a multifactorial clinical context.

Functionally, the patient′s history of excessive gum chewing is a possible contributor to repetitive mechanical overload. Studies have reported that prolonged masticatory activity can increase the electromyographic activation of the masseter muscle and alter masticatory patterns, especially in individuals predisposed to TMDs [25]. Although it has not been demonstrated to be an independent cause of TMD, this habit could act as a symptom‐amplifying factor when structural or inflammatory alterations are already present [25, 26].

In the present case, the intra‐articular space was treated with a combination of betamethasone and sodium hyaluronate, targeting synovial inflammation and joint lubrication, respectively [12]. This treatment may have contributed to the early clinical improvement; however, because treatment was multimodal, the response cannot be attributed solely to betamethasone.

Compared with arthrocentesis alone, the addition of hyaluronic acid has been associated with pain reduction and improvement in mandibular opening in certain clinical contexts [15, 27]. Sodium hyaluronate may support the viscoelastic properties of synovial fluid, improve joint lubrication, and reduce friction within the condyle–disc complex [28]. In patients with early osteoarthritis and disc displacement without reduction, its use after arthrocentesis may complement the immediate anti‐inflammatory treatment. Nevertheless, the clinical benefit observed in this patient cannot be separated from the other components of the multimodal approach [15, 28].

The concomitant use of NSAIDs during the acute phase is supported by evidence regarding the involvement of cyclooxygenases in the pathophysiology of pain and cartilage degradation in patients with TMJ osteoarthritis [12]. In addition, an occlusal stabilization splint was incorporated as an adjunct to the multimodal treatment to provide occlusal stability, reduce excessive joint loading, and limit parafunctional activity during the recovery period. Stabilizing splints have been reported to redistribute occlusal forces, reduce masseter electromyographic activity, and produce transient modifications in the condyle–fossa relationship, thereby contributing to a more stable joint environment [29, 30]. Rather than correcting the disc displacement, the appliance was intended to support symptom control and functional rehabilitation without additional mechanical overload [31].

Physical therapy and electrostimulation were provided as adjuvants to help reduce muscle hyperactivity and promote functional recovery. Manual therapy combined with therapeutic exercises has been demonstrated to reduce pain and improve the range of opening in patients with TMD, although the methodological quality of the studies is variable [9].

The clinical evolution was favorable and progressive. The patient experienced a marked reduction in pain 1 week after the procedure, suggesting an early response to the control of the intra‐articular inflammatory component. After 1 month of follow‐up, complete functional recovery was observed. This clinical course is consistent with outcomes reported for combined protocols involving arthrocentesis, intra‐articular infiltration, and adjunctive orthopedic management, particularly when treatment is initiated during the early stages of disc displacement without reduction [27, 28, 32].

The main strengths of this case report include the integration of a standardized clinical assessment with CBCT and MRI findings, the detailed and sequential description of the therapeutic approach, and the longitudinal clinical follow‐up of the patient′s response to multimodal management.

The main limitations of this case report include its single‐patient design, the relatively short 3‐month follow‐up period, which limits the assessment of long‐term clinical stability and symptom recurrence, and the inability to establish a direct causal relationship between hormonal changes, parafunctional habits, and the TMJ findings.

Although this is a case report, the rapid improvement in symptoms and the functional stability observed at the 3‐month follow‐up reinforce the importance of early, individualized, and multimodal management in young patients with joint involvement. The educational value of this case lies in the integration of clinical, CBCT, and MRI findings rather than in demonstrating the superiority of a new treatment.

## 4. Conclusion

In young patients, anterior disc displacement without reduction can coexist with structural and inflammatory findings at an early stage, suggesting a complex articular scenario in which biomechanical, inflammatory, and endocrine factors may overlap without necessarily implying direct causal relationships. The rapid resolution of pain and complete functional recovery following a multimodal approach that included arthrocentesis, intra‐articular infiltration of corticosteroids and hyaluronic acid, a stabilization splint, and complementary physical therapy highlight the importance of early and individualized interventions aimed at controlling inflammation and optimizing joint biomechanics.

## Author Contributions

Conceptualization: Daniela Chamba‐León and Wilson Daniel Bravo Torres. Methodology: Daniela Chamba‐León and Wilson Daniel Bravo Torres. Investigation: Madeleyne Priscila Serrano Toledo and Juan Diego Sabay‐Aldas. Data curation: Daniela Chamba‐León. Formal analysis: Juan Diego Sabay‐Aldas and Kevin Ochoa Suarez. Visualization: Daniela Chamba‐León and Madeleyne Priscila Serrano Toledo. Writing—original draft: Daniela Chamba‐León and Madeleyne Priscila Serrano Toledo. Supervision: Wilson Daniel Bravo Torres. Writing—review and editing: Daniela Chamba‐León, Madeleyne Priscila Serrano Toledo, Juan Diego Sabay‐Aldas, Kevin Ochoa Suarez, and Wilson Daniel Bravo Torres.

## Funding

No funding was received for this manuscript.

## Disclosure

All authors have read and approved the final version of the manuscript. Daniela Chamba‐León, the corresponding author and manuscript guarantor, had full access to all of the data in this study and takes complete responsibility for the integrity of the data and the accuracy of the data analysis.

## Ethics Statement

Ethical approval was not required for this case report because it describes a single anonymized patient, and all diagnostic and therapeutic procedures were performed as part of routine clinical care.

## Consent

Written informed consent was obtained from the patient for publication of this case report and all accompanying clinical and radiological images.

## Conflicts of Interest

The authors declare no conflicts of interest.

## Patient Perspective

The patient reported substantial pain relief after treatment and expressed satisfaction with the recovery of mandibular function. She also reported improvement in chewing comfort and daily activities after discontinuing gum chewing and following the recommended therapeutic measures.

## Supporting information


**Supporting Information** Additional supporting information can be found online in the Supporting Information section. File S1: CARE checklist.

## Data Availability

All data supporting the findings of this case report are included within the article. Additional information is available from the corresponding author upon reasonable request.
